# The prognostic value of stromal and epithelial periostin expression in human breast cancer: correlation with clinical pathological features and mortality outcome

**DOI:** 10.1186/s12885-016-2139-y

**Published:** 2016-02-12

**Authors:** P. V. Nuzzo, A. Rubagotti, L. Zinoli, S. Salvi, S. Boccardo, F. Boccardo

**Affiliations:** Academic Unit of Medical Oncology, IRCCS AOU San Martino-IST, San Martino University Hospital and National Cancer Research Institute, L.go R. Benzi 10, 16132 Genoa, Italy; Department of Internal Medicine, School of Medicine, University of Genoa, L.go R. Benzi 10, 16132 Genoa, Italy; Histopathology and Cytology Unit, IRCCS AOU San Martino-IST, San Martino University Hospital and National Cancer Research Institute, L.go R. Benzi 10, 16132 Genoa, Italy

**Keywords:** Human periostin protein, Breast neoplasms, Extracellular matrix proteins, Prognosis, Biomarkers

## Abstract

**Background:**

PN is a secreted cell adhesion protein critical for carcinogenesis. In breast cancer, it is overexpressed compared to normal breast, and a few reports suggest that it has a potential role as a prognostic marker.

**Methods:**

Tumour samples obtained at the time of mastectomy from 200 women followed for a median time of 18.7 years (range 0.5–29.5 years) were investigated through IHC with a polyclonal anti-PN antibody using tissue microarrays. Epithelial and stromal PN expression were scored independently according to the percentage of coloured cells; the 60^th^ percentile of PN epithelial expression, corresponding to 1 %, and the median value of PN stromal expression, corresponding to 90 %, were used as arbitrary cut-offs. The relationships between epithelial and stromal PN expression and clinical-pathological features, tumour phenotype and the risk of mortality following surgery were analysed. Appropriate statistics, including the Fine and Gray competing risk proportional hazard regression model, were used.

**Results:**

The expression of PN in tumour epithelial cells was significantly lower than that which was observed in stromal cells (*p* < 0.000). No specific association between epithelial or stromal PN expression and any of the clinical-pathological parameters analysed was found as it was observed in respect to mortality when these variables were analysed individually. However, when both variables were considered as a function of the other one, the expression of PN in the stromal cells maintained a statistically significant predictive value with respect to both all causes and cancer-specific mortality only in the presence of high epithelial expression levels. No significant differences in either all causes or BCa-specific mortality rates were shown according to epithelial expression for tumours displaying higher stromal PN expression rates. However, the trends were opposite for the higher stromal values and the patients with high epithelial expression levels denoted the group with the worst prognosis, while higher epithelial values in patients with lower stromal expression levels denoted the group with the best prognosis, suggesting that PN epithelial/stromal interactions play a crucial role in breast carcinogenesis, most likely due to functional cross-talk between the two compartments. On the basis of PN expression in both compartments, we defined 4 subgroups of patients with different mortality rates with the group of patients characterized by positive epithelial and low stromal PN expression cells showing the lowest mortality risk as opposed to the groups of patients identified by a high PN expression in both cell compartments or those identified by a low or absent PN expression in both cell compartments showing the worst mortality rates. The differences were highly statistically significant and were also retained after multiparametric analysis. Competing risk analysis demonstrated that PN expression patterns characterizing each of previous groups are specifically associated with cancer-specific mortality.

**Conclusions:**

Although they require further validation through larger studies, our findings suggest that the patterns of expression of PN in both compartments can allow for the development of IHC “signatures” that maintain a strong independent predictive value of both all causes and, namely, of cancer-specific mortality.

## Background

In spite of the major achievements of mammography screening and of multimodality treatments, BCa still represents the leading cause of cancer death among women in western countries [1]. While for many years treatment choices have been tailored to clinical-pathological features [2], many studies have recently focused on individual gene or protein candidates with a potential causative role in breast carcinogenesis, in the hope of identifying novel prognostic/predictive markers able to refine the information provided by clinical-pathological features [3–7].

Many of the cell abnormalities identified in solid tumours involve structural proteins. One such protein, PN, is produced and secreted by fibroblasts as a component of the ECM. This protein, which is involved in regulating intercellular adhesion [8, 9], has been recently suggested to play a relevant role in human carcinogenesis [10, 11], either through the interaction with multiple cell-surface receptors, most notably integrins [12, 13], or with the PI3-K/Akt pathway and other pathways [14, 15]. The activation of these pathways promotes cell survival, angiogenesis, invasion, metastasis, and perhaps more importantly, epithelial-mesenchymal transition of carcinoma cells [16, 17]. The overexpression of PN in cancer stroma and/or epithelium is usually associated with the most malignant phenotypes and/or with the poorest outcomes [10, 11]. To the best of our knowledge, to date only a few studies have investigated the clinical relevance of PN expression in BCa [18–20]. A statistically significant association between epithelial overexpression and poor prognosis features has been reported in two studies [18, 19] while a direct relationship between PN epithelial expression and tumour stage was described in another small study [20]. Indeed, none of the previous studies has investigated the prognostic role of PN stromal expression in BCa, though PN stromal overexpression was significantly associated with tumour aggressiveness and/or prognosis in other types of solid tumours, including lung, prostate, kidney, pancreatic, colon and ovarian cancers [10, 11].

Previous findings prompted us to conceive the present study, which was originally aimed at further exploring the prognostic value of PN expression in BCa patients.

## Methods

### Patient selection and ethical aspects

We selected a cohort of 200 patients who had a histologically confirmed diagnosis of BCa between January 1985 and November 1990; these patients were subsequently followed up at our Institute. The cohort was selected based on the availability of a corresponding serum sample drawn at the time of surgery and cryopreserved up to processing. We aimed to evaluate the prognostic value of serum levels of PN as well. The results of this second part of the project form the object of a separate paper [21]. Patients’ demography is summarised in Table 1.

**Table 1:** Main characteristic of study patients (*N* = 200)

	No. of patients (%)
Age at surgery, years	
Median (range)	58 (31–84)
Menopausal status	
Pre-menopausal	63 (31.5)
Post- menopausal	137 (68.5)
Tumour size: cm in diameter	
≤ 2	94 (47.0)
> 2	106 (53.0)
Nodal status	
Node-negative	105 (52.5)
Node-positive	95 (47.5)
ER status	
Poor (<10 % of stained cells)	42 (21.0)
Rich (≥10 % of stained cells)	158 (79.0)
PgR status	
Poor (<10 % of stained cells)	86 (43.0)
Rich (≥10 % of stained cells)	114 (57.0)
Ki-67	
Low (<14 % of stained cells)	91 (45.5)
High (≥14 % of stained cells)	109 (54.5)
HER2 status	
Negative	183 (91.5)
Positive	17 (8.5)
Phenotype^a^	
Luminal A	64 (32.0)
Luminal B (HER2-neg)	85 (42.5)
Luminal B (HER2-pos)	9 (4.5)
HER2 positive (non luminal)	8 (4.0)
Triple negative	34 (17.0)
Adjuvant systemic therapy^b^	
No	87 (43.5)
Yes	113 (56.5)

^a^definition of intrinsic subtypes of breast cancer according to [23]

^b^either chemotherapy or endocrine therapy or both

This research project was approved by the Ethical Committee of Regione Liguria, and the patients’ data were managed according to the Italian Data Protection Authority prescriptions (http://www.garanteprivacy.it).

### IHC analysis

IHC evaluations were performed using 3-μm sections of paraffin embedded TMAs. Using the Tissue–Tek Quick-Ray TM, two 2.0 mm diameter cores of tumour tissue were incorporated into a 10 × 6 (60 cores) TMA recipient block. Four TMAs were constructed containing two cores for each one of the 200 tumour blocks.

The PN (OSF-2) polyclonal rabbit antibody (Acris Antibodies, Herford, Germany) suitable for the various isoforms of PN was used at a dilution of 1:500. We used this antibody in a previous study in prostate cancer [22]. TMA sections were immune stained using the Benchmark XT automatic stainer (Ventana Medical Systems, SA Strasbourg, France). Slides were deparaffinized, and after adding high pH, heat induced, standard citrate buffer (30 min), the antibody-antigen complex was relieved using the polymeric detection system (Ventana Medical System Ultraview Universal DAB Detection Kit). A negative and a positive control were used for each staining run. The negative control consisted of performing the entire IHC procedure on adjacent sections in the absence of the primary antibody. Then, the sections were counter-stained with Gill's modified haematoxylin, cover-slipped and evaluated at 10×, 40× and 60× magnifications by two different observers (S.S & B.S.) using an Olympus multi-headed light microscope. The entire area of each tumour core was analysed.

To correlate PN expression to clinical pathological variables, the tumour phenotype was re-assessed on the same TMAs on the basis of the ER clone SP1, the PgR clone 1E2, the Ki-67 clone 30–89 and the HER2 clone 4B5b expression; IHC protocols currently adopted in the Histopathology Unit of our Institute (see Authors affiliation) and based on the use of the IHC detection system Ultra View DAB Detection Kit (Roche Ventana Medical System, Tucson, AZ, USA) were followed.

### PN, ER, PgR, KI67 and HER2 Scoring

**Fig. 1:**
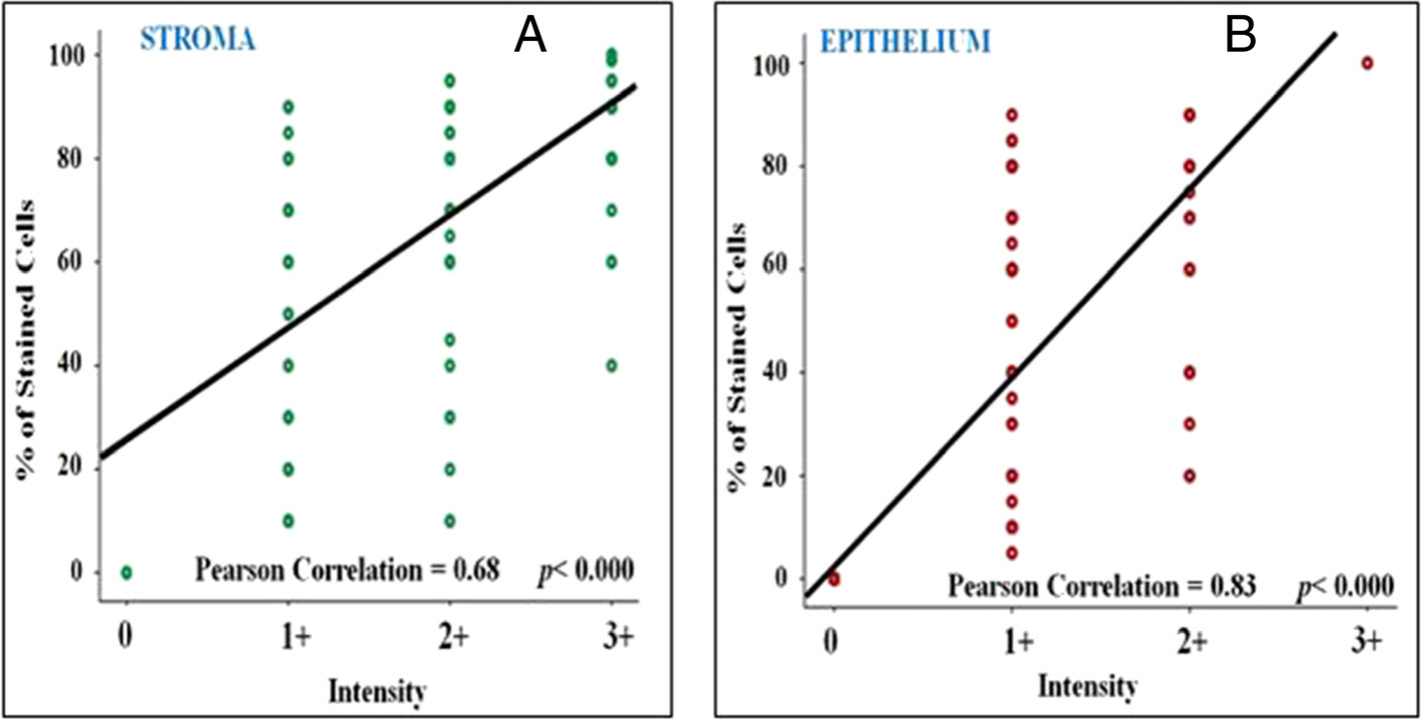
The correlation between the intensity of immunostaining and the percentage of stained cells in the tumour stromal (**a**) and epithelial (**b**) compartments

To score epithelial and stromal PN expression, we initially decided to use the scoring system IRS adopted in the previously mentioned study in prostate cancer [22]. IRS is obtained by multiplying the intensity value of immune coloration by the percentage of stained cells, and the score is applied to both epithelial and stromal cells. The intensity of staining is arbitrarily graded as: absent (0), weak (1+), moderate (2+), and strong (3+) while the percentage of stained cells is quantified as negative (0 % of positive cells), 1+ (<10 % positive cells), 2+ (10–50 % of positive cells), 3+ (51–80 % of positive cells), and 4+ (>80 % of positive cells). However, we realized that there was still a certain inter-observer variation in scoring the intensity of immune coloration and because a strong relationship was found between the staining intensity and the number of coloured cells, both in the epithelial and in the stromal compartment (Fig. 1), we decided that scoring PN expression exclusively as a function of the percentage of coloured cells might be easier and more reproducible.

For ER, PgR and Ki67 scoring, the nuclear staining was evaluated through the percentage of nuclear neoplastic area on the total area analysed, using the image analyser Leica QWin software connected to a light microscope Leica DMLA and using a 40× magnification. A Ki-67 proliferation index threshold of 14 % was used to distinguish tumours with low (<14 %) or high (≥14 %) proliferative fractions. Relative to steroid hormone receptor status, tumours were defined as ER a/o PgR poor (no nuclear staining or nuclear staining up to 9 % of cells) or as ER a/o PgR rich (nuclear staining equal to 10 % or more of cells). For HER2, tumour cores were assessed using the anti-HER2/neu (4B5) rabbit monoclonal primary antibody (Roche Ventana Medical System, Tucson, AZ, USA) and, as recommended, only HER2 3+ tumours were regarded as positive; HER2 2+ tumours were regarded as positive only if a FISH assay (Vysis LSI HER2/neu spectrum orange/CEP 17 spectrum green probe, Abbott molecular, Abbott Park, Illinois, USA) demonstrated HER2 gene amplification. Based on the 4 variables, tumours were grouped into five major phenotypes, including luminal A and luminal B types (i.e., B-like HER2 negative and B-like HER2 positive, respectively), HER2-like and triple negative phenotypes [23].

## Statistical analysis

### The correlation between PN expression in the epithelial or stromal cells and clinical-pathological variables

The *t*-test was applied to compare the mean values (SE) of epithelial PN expression with stromal PN expression values. The chi-square test was used to analyse the distribution of PN phenotypes within the different tumour subgroups, constructed on categorical variables.

The correlation between either epithelial or stromal PN expression and clinical-pathological variables was investigated with the Pearson test.

### The correlation between PN expression at the epithelial/stromal level with all causes and BCa-specific mortality

Mortality data were obtained by consulting the patients’ flow charts. Information about the women who were lost to follow-up was obtained by consulting the local Mortality Registry or the registry offices of the patients’ place of residence. All events that occurred by the deadline of December 31^st^, 2013 were recorded and causes of death were reported.

Mortality curves were constructed through the cumulative incidence function estimate by the Kaplan-Meier method and compared using the log-rank test [24].

**Fig. 2:**
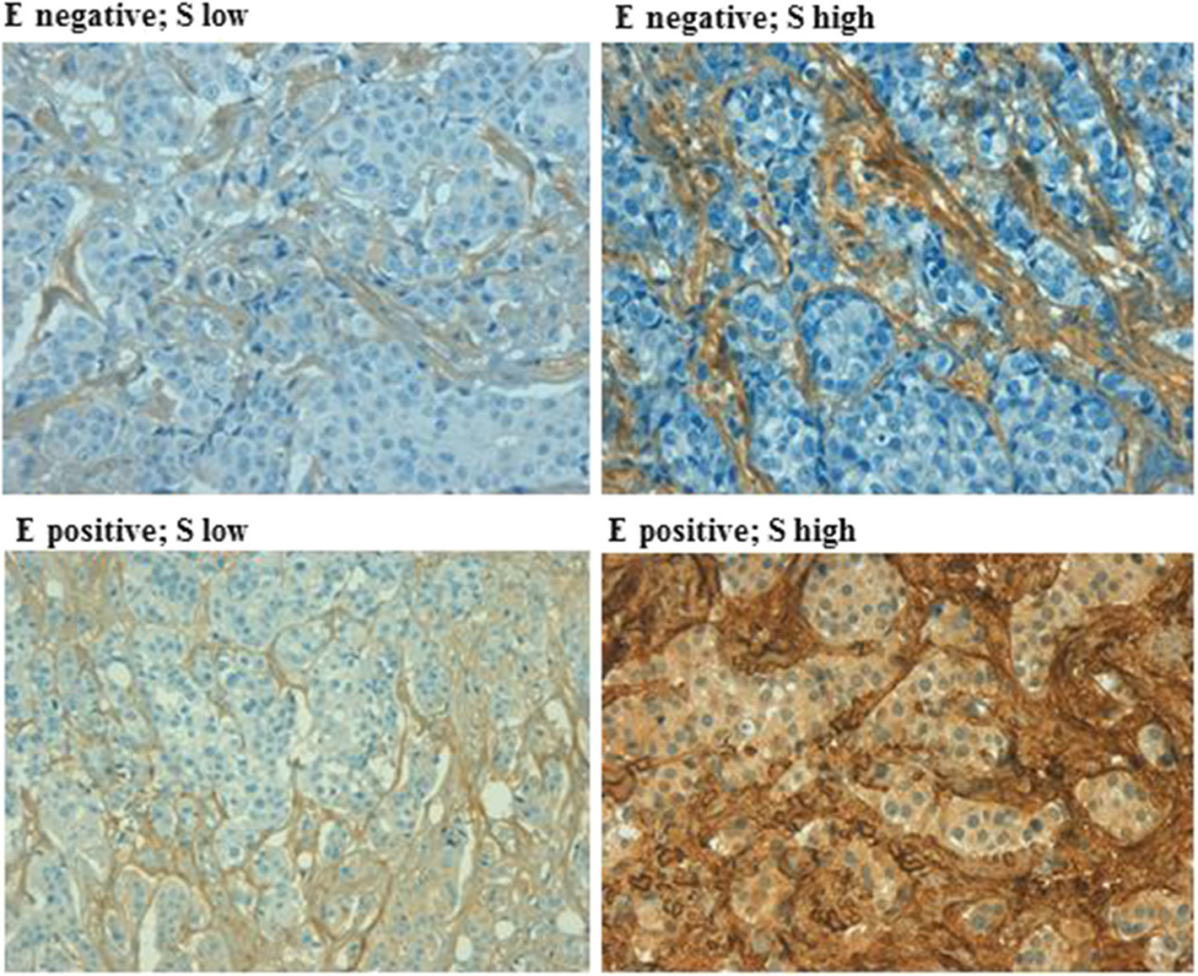
Tumour specimens corresponding to the 4 PN phenotypes identified based both on epithelial and stromal expression rates (see text). Negative: 0 % of stained cells; positive: at least 1 % of stained cells; low: less than 90 % of stained cells; high: more or equal to 90 % of stained cells

Stromal PN expression was first analysed in univariate models utilizing the median percentages of immune stained cells chosen as an arbitrary cut-off. The median percentage value of stromal PN immune coloration was 90 %. Because the median percentage value of epithelial PN immune coloration was 0 %, patients were broken down according to the 60^th^ percentile, which corresponded to 1 %. The use of this cut-off in practice implied separating epithelial PN negative cells from epithelial PN positive cells. Based on the expression rates of coloured epithelial and stromal cells, 4 distinct PN tumour phenotypes could be arbitrarily identified: 1) epithelial PN positive tumours also expressing high PN stromal values (≥90 %), 2) epithelial PN positive tumours expressing low PN stromal values (i.e., <90 %), 3) epithelial PN negative tumours expressing high PN stromal levels, and 4) epithelial negative PN tumours expressing low PN stromal levels (Fig. 2). To evaluate the independent role of PN expression in either stromal or in epithelial tumour cells and that of the 4 phenotypes identified based both on epithelial and stromal expression rates, Cox proportional hazards models were fitted to all causes and BCa-specific mortality data [25]. The cumulative incidence function was used to describe cause-specific mortality, and the Fine and Gray’s test was used to investigate the cause-specific mortality differences [26]. Mortality risks were expressed as HR estimates and their 95 % confidence intervals CIs were also calculated. All *p* values were two-tailed. The IBM software Statistical Package for Social Sciences (SPSS) version 21.0 for Windows (SPSS Inc. Chicago, Illinois, USA) and STATASE11 were used for data analysis.

## Results

### PN expression in the epithelial and stromal cells

Distinct stromal and epithelial staining characteristics allowed for the evaluation of PN staining in the selected TMAs. Both in epithelial and in stromal cells, PN was expressed mainly in the cytoplasm, which showed a diffuse granular tan coloration. Of the 200 tissue specimens, 104 (52 %) displayed ≥80 %, 64 (32 %) 51–79 %, and 29 (14.5 %) 10–50 % stromal cell PN staining while only 3 specimens (1,5 %) showed no staining for PN in cells. As previously mentioned, the median percentage value of stromal cells expressing PN was 90 %. The expression of PN in tumour epithelial cells was significantly lower than in stromal cells (*p* < 0.000). In fact, 120 (60 %) of the 200 tissue specimens showed no epithelial PN staining at all; 34 (17 %) displayed 10–50 % and 38 out of 200 (23 %) showed 51–80 % coloured cells while only 8 specimens showed ≥80 % coloured cells. Notably, PN expression in stromal cells significantly correlated with PN expression in epithelial cells (Pearson correlation test: *p* < 0.000; data not shown).

### The association between PN expression in epithelial and/or stromal cells and clinical-pathological variables

No specific correlation of epithelial or stromal PN expression with any of the clinical-pathological variables (age at surgery, menopausal status, tumour size, nodal status, ER status, PgR status, proliferative activity, HER2 expression/amplification and tumour phenotype according to the four variables) was found (Table 2). Accordingly, no significant correlation of the 4 epithelial/stromal PN phenotypes with clinical pathological variables was observed (data not shown).

**Table 2:** Correlation between either epithelial or stromal PN expression and clinical-pathological variables

	Epithelial Expression (% cells stained)			Stromal Expression (% cells stained)		
	Negative^a^	Positive^a^	*p*=	Low^a^	High^a^	*p*=
	*n* = 120	*n* = 80		*n* = 99	*n* = 101	
Median age at surgery, years						
≤ 58	64 (53.3 %)	39 (48.8 %)	0.6	57 (57.6 %)	46 (45.5 %)	0.1
> 58	56 (46.7 %)	41 (51.2 %)		42 (42.4 %)	55 (54.5 %)	
Menopausal status						
Pre-menopausal	41 (34.2 %)	22 (27.5 %)	0.3	35 (35.4 %)	28 (27.7 %)	0.3
Post-menopausal	79 (65.8 %)	58 (72.5 %)		64 (64.6 %)	73 (72.3 %)	
Tumour size: cm in diameter						
≤ 2	61 (50.8 %)	33 (41.3 %)	0.2	51 (51.5 %)	43 (42.6 %)	0.2
> 2	59 (49.2 %)	47 (58.7 %)		48 (48.5 %)	58 (57.4 %)	
Nodal status						
Node-negative	60 (50.0 %)	45 (56.3 %)	0.4	50 (50.5 %)	55 (54.5 %)	0.6
Node-positive	60 (50.0 %)	35 (43.7 %)		49 (49.5 %)	46 (45.5 %)	
ER status						
Poor	29 (24.2 %)	13 (16.3 %)	0.2	23 (23.2 %)	19 (18.8 %)	0.4
Rich	91 (75.8 %)	67 (83.7 %)		76 (76.8 %)	82 (81.2 %)	
PgR status						
Poor	50 (41.7 %)	36 (45.0 %)	0.6	46 (46.5 %)	40 (39.6 %)	0.3
Rich	70 (58.3 %)	44 (55.0 %)		53 (53.5 %)	61 (60.4 %)	
Ki-67						
Low	53 (44.2 %)	38 (47.5 %)	0.6	44 (44.4 %)	47 (46.5 %)	0.7
High	67 (55.8 %)	42 (52.5 %)		55 (55.6 %)	54 (53.5 %)	
HER2 status						
Negative	110 (91.7 %)	73 (91.3 %)	0.9	88 (88.9 %)	95 (94.1 %)	0.2
Positive	10 (8.3 %)	7 (8.7 %)		11 (11.1 %)	6 (5.9 %)	
Phenotype^b^						
Luminal A	39 (32.5 %)	25 (31.3 %)	0.6	29 (29.3 %)	35 (34.7 %)	0.7
Luminal B (HER2-neg)	47 (39.2 %)	38 (47.5 %)		41 (41.4 %)	44 (43.6 %)	
Luminal B (HER2-pos)	5 (4.2 %)	4 (5.0 %)		6 (6.1 %)	3 (3.0 %)	
HER2 positive (non luminal)	5 (4.2 %)	3 (3.7 %)		5 (5.1 %)	3 (3.0 %)	
Triple negative	24 (20.0 %)	10 (12.5 %)		18 (18.2 %)	16 (15.8 %)	
Adjuvant systemic therapy^c^						
No	48 (40.0 %)	39 (48.8 %)	0.2	43 (43.4 %)	44 (43.6 %)	0.9
Yes	72 (60.0 %)	41 (51.2 %)		56 (56.6 %)	57 (56.4 %)	

^a^Negative: 0 % of stained cells; positive: at least 1 % of stained cells; low: less than 90 % of stained cells; high: more or equal to 90 % of stained cells

^b^definition of intrinsic subtypes of breast cancer according to [23]

^c^either chemotherapy or endocrine therapy or both

### The correlation between PN expression in epithelial and/or stromal cells with all causes and BCa-specific mortality

**Fig. 3:**
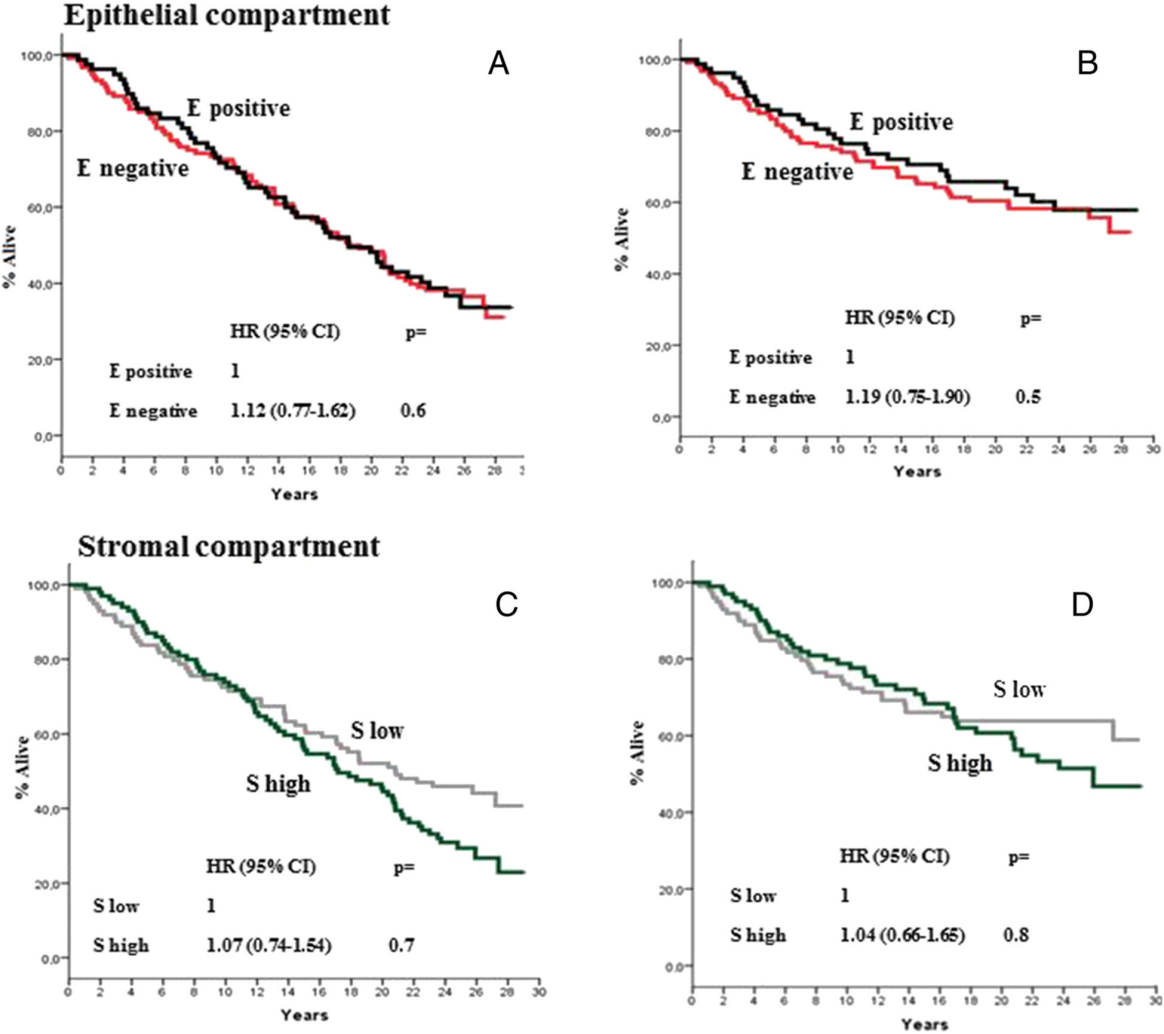
All causes (**a** and **c**) and BCa-specific mortality (**b** and **d**) of study patients as a function of epithelial and stromal PN expression. Epithelial PN expression was analysed using the 60^th^ percentile of immune stained cells, corresponding to 1 % as an arbitrary cut-off. Stromal PN expression was analysed using the median percentage of immune stained cells, corresponding to 90 % as an arbitrary cut-off. All causes and BCa-specific mortality comparisons were adjusted by age, menopausal status, tumour size, nodal status, and adjuvant systemic therapy: see text for further explanations. Negative: 0 % of stained cells; positive: at least 1 % of stained cells; low: less than 90 % of stained cells; high: more or equal 90 % of stained cells. HR: hazard ratio, 95 % CI: 95 % confidence interval

**Fig. 4:**
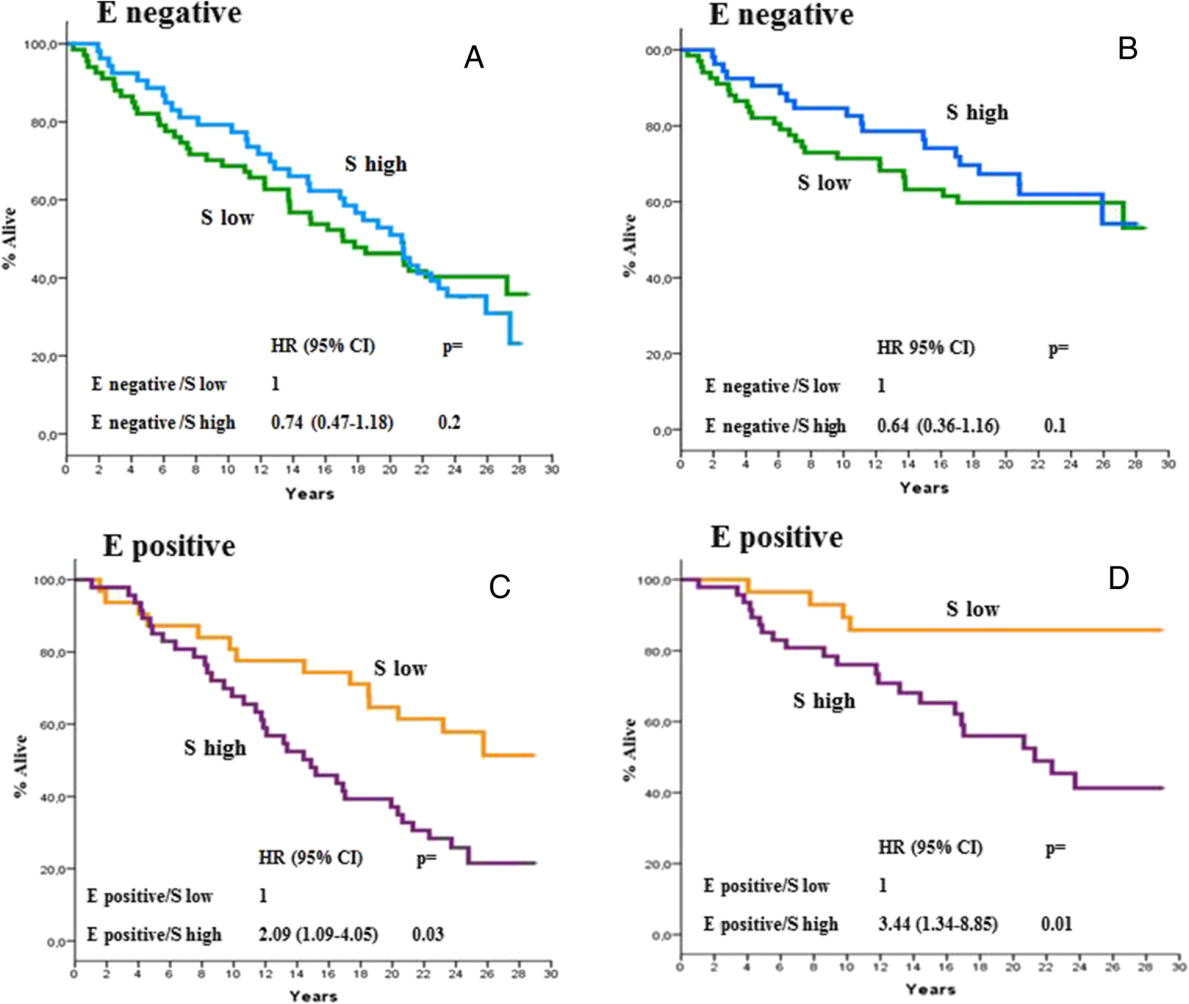
All causes (**a** and **c**) and BCa-specific mortality (**b** and **d**) of study patients according to PN epithelial expression as a function of stromal PN expression. Epithelial PN expression was analysed using the 60^th^ percentile of immune stained cells, corresponding to 1 % as an arbitrary cut-off. Stromal PN expression was analysed using the median percentage of immune stained cells, corresponding to 90 % as an arbitrary cut-off. All causes mortality and BCa-specific mortality comparisons were adjusted by age, menopausal status, tumour size, nodal status, and adjuvant systemic therapy: see text for further explanations. Negative: 0 % of stained cells; positive: at least 1 % of stained cells; low: less than 90 % of stained cells; high: more or equal 90 % of stained cells. H: hazard ratio, 95 % CI: 95 % confidence interval

**Fig. 5:**
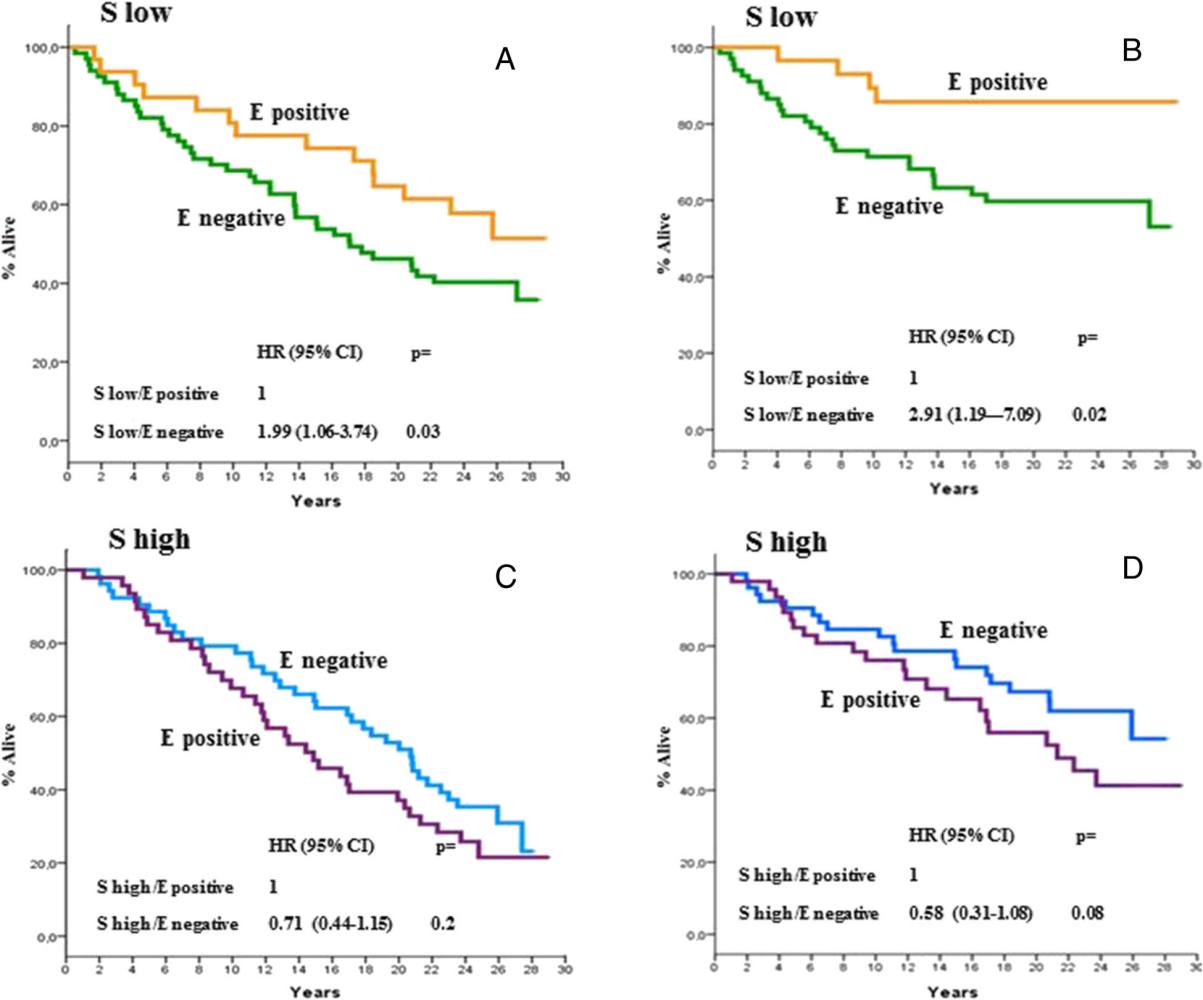
All causes (**a** and **c**) and BCa-specific mortality (**b** and **d**) of study patients according to PN stromal expression as a function of epithelial PN expression. Epithelial PN expression was analysed using the 60^th^ percentile of immune stained cells, corresponding to 1 % as an arbitrary cut-off. Stromal PN expression was analysed using the median percentage of immune stained cells, corresponding to 90 % as an arbitrary cut-off. All causes and BCa-specific mortality comparisons were adjusted by age, menopausal status, tumour size, nodal status, and adjuvant systemic therapy: see text for further explanations. Negative: 0 % of stained cells; positive: at least 1 % of stained cells; low: less than 90 % of stained cells; high: more or equal 90 % of stained cells. HR: hazard ratio, 95 % CI: 95 % confidence interval

**Fig. 6:**
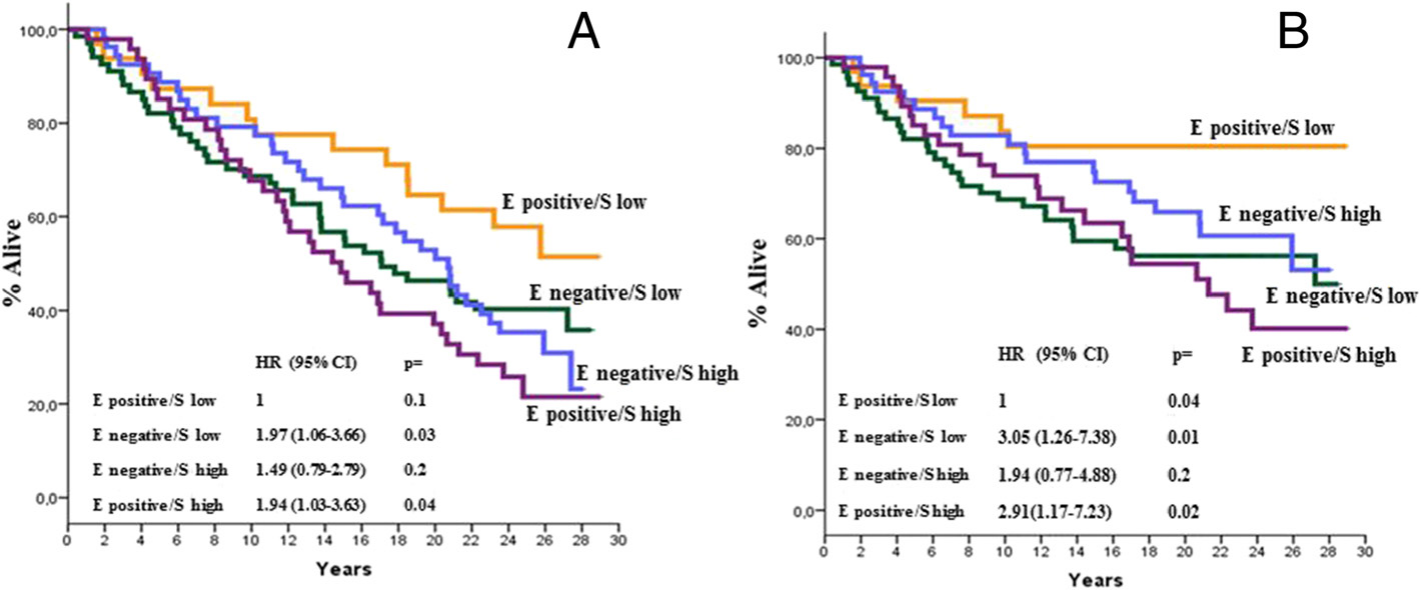
All causes (**a**) and BCa-specific mortality (**b**) of study patients according to the four distinct PN tumour phenotypes identified on the basis of both epithelial and stromal PN expression. Epithelial PN expression was analysed using the 60^th^ percentile of immune stained cells, corresponding to 1 % as an arbitrary cut-off. Stromal PN expression was analysed using the median percentage of immune stained cells, corresponding to 90 % as an arbitrary cut-off. All causes and BCa-specific mortality comparisons were adjusted by age, menopausal status, tumour size, nodal status, and adjuvant systemic therapy: see text for further explanations. Negative: 0 % of stained cells; positive: at least 1 % of stained cells; low: less than 90 % of stained cells; high: more or equal 90 % of stained cells. HR: hazard ratio, 95 % CI: 95 % confidence interval

At a median follow-up time of 18.7 years (range,0.5–29.5), 126 deaths were recorded, of which 79 were BCa-related. As previously mentioned, the correlation between PN expression with all causes and BCa-specific mortality was explored in univariate models. As is shown in Fig. 3a, b, c, and d, no correlation was found when PN expression in stromal or epithelial cells was analysed on an individual basis. However, as is shown in Figs. 4a, b, c and d and 5a, b, c and d, there were distinct interactions between PN expression in the two compartments and either all causes or BCa-specific mortality when the two variables (i.e., epithelial or stromal expression) were analysed as one as a function of the other. In fact, there were no substantial differences in all causes or BCa-specific mortality rates according to PN stromal expression as a function of epithelial expression for epithelial negative tumours (Fig. 4a & b). In contrast, a statistically significant association between stromal PN expression and both all causes and BCa-specific mortality was observed for epithelial positive tumours (Fig. 4c & d) even after adjusting comparisons by age, menopausal status, tumour size, nodal status and adjuvant systemic therapy, i.e., those variables listed in Table 1 showing to predict mortality in univariate models (data not shown). The analysis of the correlation of PN epithelial expression as a function of stromal expression with either all causes or BCa-specific mortality yielded specular results. In fact, while no significant differences in either all causes or BCa-specific mortality rates were shown according to epithelial expression for tumours displaying higher stromal PN expression rates (Fig. 5c & d), a statistically significant difference favoured PN epithelial positive tumours displaying lower PN stromal expression levels (Figs. 5a & b), again after adjusting for age, menopausal status, tumour size, nodal status and adjuvant systemic therapy. Comparable trends emerged when the mortality analysis was extended to the 4 PN epithelial/stromal phenotypes previously identified. Figure 6a shows that in fact the 4 groups have different probabilities of dying, the best outcome favouring the patients with epithelial positive and low stromal PN expression rates and the worst outcome for patients with either epithelial positive and high stromal PN expression rates or epithelial negative and low stromal expression rates. The results relative to BCa-specific mortality were almost comparable (Fig. 6b). In both cases, the comparisons were adjusted for the covariates previously mentioned.

**Fig. 7:**
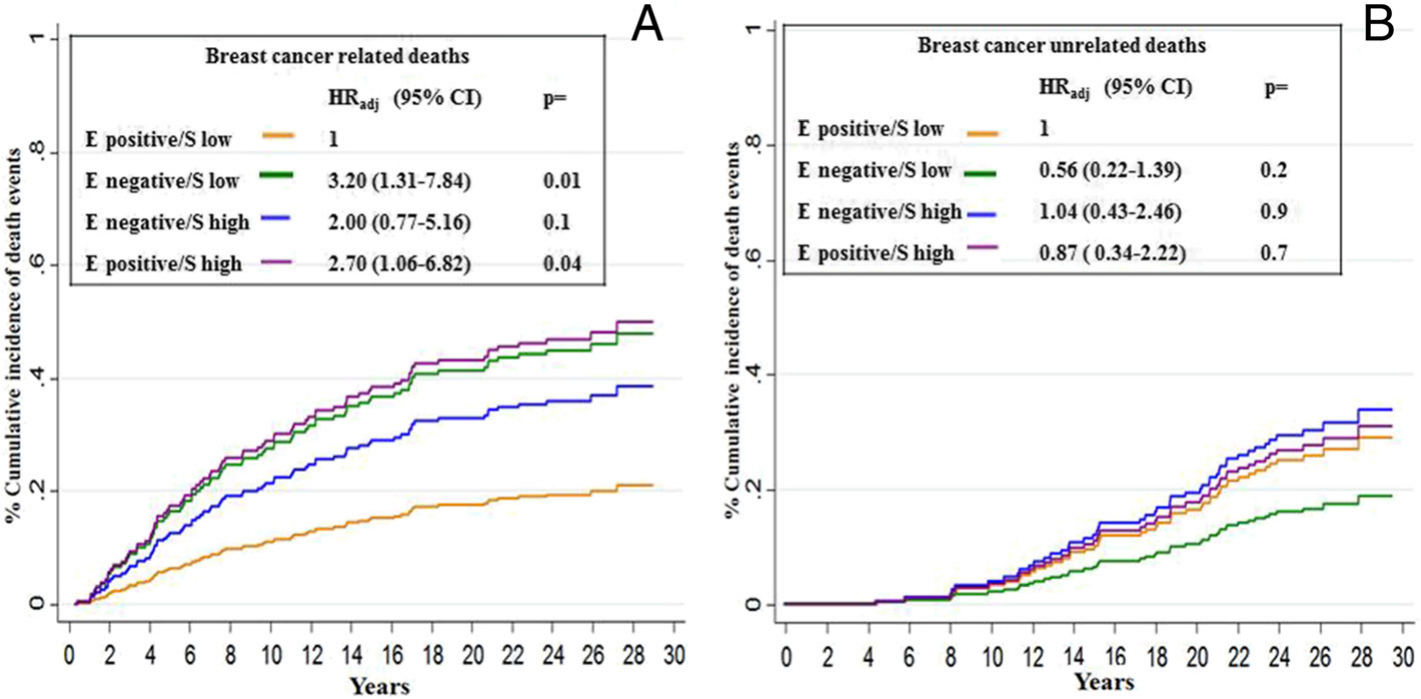
Cumulative incidence of death at 30 years from surgery according to both epithelial and stromal PN expression (**a**: Breast cancer related deaths; **b**: Breast cancer unrelated deaths) Epithelial PN expression was analysed using the 60^th^ percentile of immune stained cells, corresponding to 1 % as an arbitrary cut-off. Stromal PN expression was analysed using the median percentage of immune stained cells, corresponding to 90 % as an arbitrary cut-off. Cumulative incidence of death comparisons were adjusted by age, menopausal status, tumour size, nodal status, and adjuvant systemic therapy: see text for further explanations. Negative: 0 % of stained cells; positive: at least 1 % of stained cells; low: less than 90 % of stained cells; high: more or equal 90 % of stained cells. HR: hazard ratio, 95 % CI: 95 % confidence interval

The association between each one of the 4 epithelial-stromal PN phenotypes and mortality with respect to the cause of death was further and more accurately investigated through competing risk analysis (Fig. 7a & b). The curves show that PN phenotypes do not appear to correlate with breast cancer unrelated deaths and, in particular, that there is no difference at all relative to BCa unrelated mortality between the phenotype with a better prognosis and those showing the poorest outcomes. In contrast, a statistically significant correlation of PN epithelial/stromal phenotypes with BCa-specific mortality was observed; in particular, a strict relationship between the PN phenotype and increasing probability of death was observed. In fact, the lowest probability was observed for the women characterized by the most favourable phenotype (epithelial positive, low stromal expression) while the highest probabilities were observed for the women characterized by the less favourable PN phenotypes (epithelial negative, low stromal expression; epithelial positive, high stromal expression).

## Discussion

### PN expression in the epithelial and stromal cells

We have shown that PN is mostly expressed by stromal cells where it appears to be localized mainly in the cytoplasm. In fact, of the 200 tissue specimens included into our cohort, 104 (52 %) displayed ≥80 %, 64 (32 %) 51–79 %, and 29 (14.5 %) 10–50 % stromal cell PN staining while only 3 specimens (1,5 %) showed no staining for PN in cells. This finding is an obvious consequence of the fact that PN is one of the major components of the ECM and is consistent with previous observations by us and other investigators in the great majority of solid tumours [10, 11]. A prevalent expression of PN in the stroma of tissue samples selected for previously mentioned studies in BCa was also reported; however, neither Puglisi’s group [18] nor the Chinese group [19] provide detailed information about PN stromal expression and both studies, as already mentioned before, do not take into account stromal expression in their attempt to correlate PN expression with clinical pathological variables or clinical outcome. No adequate information on PN expression and localization is provided by Zhang et al. [20]. We also found that PN is expressed in 40 % of epithelial cells. Our data are comparable with those reported by Puglisi et al. [18], who observed epithelial staining in 57 % of epithelial cells, and those reported by Xu et al. [19], who showed PN epithelial staining in 30 % of cells. Taken together, our findings and those reported in the literature confirm that in BCa tissues PN is mostly and highly expressed in stromal cells but that the protein is also expressed in 30–60 % of epithelial tumour cells and that, in both cell compartments, it appears to localize mainly in the cytoplasm [18, 19]. These findings, consistent with previous observations in other solid tumours [10, 11], corroborate the assumption that PN might play a major role in carcinogenesis.

### The correlation between PN expression with clinical-pathological variables

As previously reported in the results session, no specific correlation between either epithelial or stromal PN expression with any of the clinical-pathological variables considered by us (age at surgery, menopausal status, tumour size, nodal status, ER status, PgR status, HER2 expression/amplification, proliferative activity, tumour phenotype defined on the basis of the last 4 variables) was found (Table 2). Accordingly, no correlation with clinical pathological variables was found after grouping tumour samples based on the actual pattern of epithelial/stromal expression (data not shown). As previously mentioned, neither Puglisi’s [18] nor Xu’s group [19] analysed the putative correlation of PN expression in stromal cells with clinical pathological variables, though both groups showed that high PN expression was commonly found in tumour stroma; however, they only considered epithelial expression for their analysis. This fact, in our opinion, might represent a major limitation of their studies. There is in fact mounting evidence that epithelial/stromal interactions are likely crucial for the role played by PN in tumour progression through a type of functional cross-talk between the two compartments [21, 27–29]. Beyond previous considerations, we can only compare our results and those of the above mentioned studies with respect to PN expression in the epithelial cells. Puglisi et al. [18] found a significant association between PN cytoplasmic epithelial expression and tumour size, PgR status, VEGF-A and VEGFR-1, and a significant association of nuclear PN expression and tumour size, ER and PgR status, VEGF-A, VEGFR-1 and VEGFR-2. To our knowledge, these investigators are the only ones to demonstrate the nuclear expression of the protein in epithelial cells and this further complicates the comparison of their findings with our own and those reported by the other investigators. Puglisi’s findings are not consistent with the lack of any correlation between epithelial PN expression and the clinical-pathological findings observed in our study, though we did not include variables such as VEGF and its receptors [18]. We do not have an explanation for this fact; however, though both studies included comparable numbers of patients and the percentages of coloured cells was also comparable, in our study we have taken into account the percentage of coloured cells rather than the staining intensity as was done by Puglisi and co-workers [20]. Our findings also do not fit with those reported by Xu et al. [19], as in our study, epithelial PN positivity per se was not associated with any of the clinical pathological variables. Again, it is not possible to provide an explanation for the differences observed. However, it may not be trivial to underline the aspects that differentiate the two cohorts on study. In fact, the Chinese cohort was much larger than our own; moreover, there were significant differences in tumour stage at diagnosis (approximately 26 % of Chinese patients had distant metastases at diagnosis) and tumour phenotype (in particular, unusual rates of HER2 positive and triple negative tumours were recorded among Chinese patients). Genetic and epigenetic differences that can provide an explanation for the phenotypic differences between our cohort and the Chinese cohort might play a role in explaining the different correlation of PN epithelial expression with tumour phenotype in the two cohorts. Zhang et al. [20] described only a trend for PN expression to increase from stage I to stage IV tumours; however, they do not report on the putative correlation of PN expression with any clinical pathological variable, thus rendering it impossible to compare our findings with their own.

### The correlation of PN expression with all causes and BCa-specific mortality

As previously mentioned, Puglisi et al. [18] did not analyse clinical outcome according to PN expression, while Zhang et al. [20] observed that PN expression was correlated with tumour stage at diagnosis. Only Xu’s study has previously evaluated the correlation of PN epithelial expression and BCa-specific mortality, showing that high epithelial PN expression was significantly associated with a higher mortality risk. In our study, a strong correlation between positive epithelial PN expression and both all causes and BCa-specific mortality was also found but only in patients also characterized by a low stromal expression of the protein (Figs. 4, 5 and 6). The trends shown in Fig. 6a & b were confirmed by competitive risks analysis. This analysis demonstrated a strong correlation of each PN epithelial/stromal PN phenotype with BCa-related deaths, suggesting a specific role for PN expression and interaction according to its compartmentalization in affecting BCa lethality. The lack of any association of PN expression with clinical pathological variables and the maintenance of statistically significant trends observed after adjusting for the co-variates predictive in univariate models confirm the independent prognostic value of PN staining and suggest that different biological mechanisms than those merely correlated with the clinical pathological features at diagnosis might play a more crucial role in the later phases of the natural history of the disease. In a BCa murine model, Malanchi et al. [27] demonstrated that stromal PN is crucial for metastatic colonisation through the interaction with epithelial stem cells (which were also found by these investigators to overexpress PN) at the metastatic niche level. Tumour cells may stimulate or send a signal to the surrounding tissues to produce PN at the ECM level. A return signal may also exist. These biological premises might help in interpreting the results of our study where no strong association between either epithelial or stromal PN expression and mortality was observed when these variables were analysed on an individual basis while a significant association between mortality and different epithelial/stromal PN expression patterns was found. The possibility of defining groups with hugely different outcomes on the basis of the pattern of PN expression in both the epithelial and stromal compartments was already demonstrated by our group in prostate cancer [22]. Here again we were able to demonstrate that stromal expression could display different prognostic implications in the patients depending on protein epithelial expression [22]. In our previous study on prostate cancer, the type of interaction between epithelial and stromal PN expression appears to differ from that observed in the present study. However, again we should take into adequate account that different criteria have been used to categorize PN expression in the two studies (IRS, i.e., a score obtained by multiplying the percentage of coloured cells by the degree of staining intensity in the study on prostate cancer and the percentage of coloured cells in the present study) and, above all, that the intrinsic biologic differences correlated with tumour type might not allow for the comparison of the results achieved in the two studies. In fact, it is well known that different PN isoforms are expressed by different tumours and are most likely involved in carcinogenetic processes [30, 31], though, to our knowledge, information about PN isoforms specifically involved in prostate or breast cancer is currently unknown. Clearly, our findings should be regarded as merely exploratory and, as such, they should be taken with caution because they might present several limitations related to the retrospective nature of our study and methodological issues. This was, in fact, a retrospective cohort study performed on a relatively small patient cohort. Both conditions might represent a bias and affect the statistical power of the study as well as the possibility to perform appropriate sub-group comparisons. Moreover, our women may not represent the current average population of women newly diagnosed with BCa [32], and therapeutic standards and attitudes have also been changing over time. In fact, our patient cohort was referred to us between 1985 and 1990 and, therefore, show a relatively high (approximately 50 %) prevalence of women with tumours >2 cm in size a/o with nodal involvement. For this reason, transferring our findings to current clinical situations might also be difficult. The differences in patient populations and follow up duration might also render it difficult to compare our findings with the few ones previously reported in the literature [18, 19]. Other aspects that might bias the comparison of our findings with those previously reported in the literature are likely to be related to methodological issues. While the same polyclonal rabbit antibody was used in our study and in those by Puglisi et al. [18] and by Xu et al. [19], a different scoring method was used by Puglisi’s group based exclusively on the intensity of coloration. Though we have demonstrated a direct relationship between staining intensity and the percentage of coloured cells (Fig. 1), there is no doubt that determining staining intensity is much more subject to individual interpretation, especially in the absence of referee standards. However, our choice to score PN expression only on the basis of the percentage of coloured cells might be questioned, and indeed it might not represent the most appropriate scoring method, especially relative to stromal cells. In fact, we realize that it might not always be easy to distinguish between specific staining of the cellular component of the stroma from the non-specific staining of PN that accumulates in the extracellular matrix. The PN expression at the cellular level in the stroma might not be appropriate even from the biological point of view because the PN secreted in the extracellular matrix is not “biologically inert” but rather contributes to the functional role of the protein. While it is intuitive that the expression of PN in the extracellular matrix can be “quantified” only on the basis of staining intensity, it might be reassuring that the coefficient of correlation between the percentage of coloured cells in the stroma and the intensity of immune staining did not change when the intensity of the immune staining was recalculated on the basis of the intensity observed both in the cellular and in the extracellular component of the stroma (c:0.97; *p* < 0.000).

## Conclusions

In conclusion, we demonstrated that PN is expressed mainly in tumour stromal cells compared to epithelial cells and that PN expression in both compartments appears to be a biological requirement to define different prognostic subgroups of BCa. Multiparametric analysis confirms that PN expression has an independent prognostic value with respect to the clinical-pathological variables that are commonly used to define patient prognosis. The prognostic role of PN expression and compartmentalization appears to be specific for BCa-related mortality and is maintained up to 30 years after breast surgery. The identification of the specific isoforms involved in breast cancer might be of great help, not only to better elucidate the mechanisms involved in the compartmental interactions, which are thought to be crucial in driving tumour progression, but also to increase the prognostic and predictive accuracy of PN staining. As previously stated, our findings should be regarded as merely exploratory and, as such, they should be taken with caution. Nonetheless, they warrant IHC methodological standardization and further validation of the potential usefulness of PN as a prognostic marker in BCa through larger prospective studies should be investigated.

## Abbreviations

BCa: Breast cancer
CIs: Confidence intervals
ECM: Extracellular matrix
ER: Oestrogen receptor
HR: Hazard ratio
IHC: Immunohistochemical
IRS: Immune reactive score
PgR: Progesterone receptor
PN: Periostin
SE: Standard error
TMAs: Tissue microarrays
